# Sex differences in alcohol-induced transcriptional changes in the nucleus accumbens depend on sex chromosome complement and gonadal type

**DOI:** 10.21203/rs.3.rs-10093623/v1

**Published:** 2026-06-23

**Authors:** Kiara Ream, Ezra P. Eccles, Delainey G. Lewis, D’Erick Underwood, Anna K. Radke

**Affiliations:** Department of Psychology and Center for Neuroscience and Behavior, Miami University, Oxford, OH, USA

**Keywords:** sex differences, RNASeq, Four Core Genotypes mice, intermittent access, alcohol, nucleus accumbens

## Abstract

**Background:**

Studying sex differences in alcohol drinking behaviors in rodents can reveal molecular and physiological differences that may be relevant to addiction. Prior work with the Four Core Genotypes (FCG) mouse line suggests that sex chromosome complement and gonadal type contribute to ethanol consumption in male vs. female mice. Here, we confirmed an effect of sex chromosome complement on alcohol intake in Sry+ male mice using an intermittent access two-bottle choice model.

**Methods:**

Mice consumed 20% ethanol or water for 13 sessions over 4.5 weeks. Three days after drinking, tissue from the nucleus accumbens was collected and analyzed using RNASeq.

**Results:**

Robust transcriptional changes were identified in male (XY/Sry+) mice drinking ethanol vs. water but not in females (XX/Sry−). There were also limited effects of gonad type (Sry− vs. Sry+) and sex chromosome complement (XX vs. XY) on differential gene expression, suggesting that neither of these factors serves as the primary driver of the transcriptional response to alcohol.

**Conclusions:**

Both alcohol drinking and RNASeq analyses demonstrate that sex chromosome complement and gonad type interact to produce distinct behavioral and molecular profiles in males and females.

## 1. Background

Studies of sex differences in animal models of alcohol drinking have revealed important differences between males and females that could inform treatment strategies and the development of novel therapeutics. For example, alcohol intake is consistently higher in female vs. male rodents across a range of drinking paradigms. Further, females are more likely to consume and operantly respond for alcohol despite the presence of a negative consequence [1–4]. These behavioral patterns are the product of sexually dimorphic biological factors. Ovarian hormones, particularly estrogens, promote alcohol consumption in females [5,6] while testosterone tends to suppress intake in males [7]. Sex chromosome complement has also been shown to influence consumption, preference, and aversion-resistance [8–10].

The Four Core Genotypes (FCG) mouse model is a valuable tool for studying the independent and interactive influences of gonadal hormones and sex chromosome complement on alcohol drinking and the effects of alcohol exposure [11]. Because the *Sry* gene is not inherited through the Y chromosome, gonadal and chromosomal sex are dissociated in FCG mice. Both the XY and XX chromosome complements are found in mice with testes (Sry+) and mice with ovaries (Sry−), resulting in four genotypes. We have previously used these mice to demonstrate that XY with testes (Sry+) consume more alcohol during a limited access “Drinking in the Dark” procedure and demonstrate resistance to quinine aversion vs. XX/Sry+ mice [8–10]. We have also observed sex chromosome effects on alcohol preference and the alcohol deprivation effect using a continuous access drinking task [8–10]. These studies also revealed important gonadal influences, wherein consumption of water and ethanol was consistently higher in mice with ovaries (*Sry*−).

The current study had two main goals. First, we aimed to extend our investigations of alcohol drinking behaviors in FCG mice to a 24-h intermittent access two-bottle choice procedure [12]. This drinking task has frequently been used by our lab and others to study escalated alcohol consumption in mice and rats [13–15]. We predicted that the effects observed with the limited access model would be replicated with intermittent access, given that both procedures involve repeated cycles of exposure and withdrawal that produce escalation. Second, we examined how gonadal and chromosomal sex interact with alcohol exposure to affect transcriptional profiles in the nucleus accumbens (NAc) following alcohol drinking. Prior studies examining differential gene expression following alcohol exposure in this region have revealed changes in genes related to synaptic transmission, neurotransmitter metabolism (including dopamine, GABA, and glutamate), transcriptional regulation, and circadian rhythms in male rodents [16–20]. Another study in female mice used chemogenetic stimulation to reduce binge-like drinking and found that genes associated with this treatment were primarily related to neuroimmune functions and structural plasticity [21]. Targeting of biological pathways identified by these types of bioinformatic analyses has also successfully decreased alcohol consumption [21,22], demonstrating the utility of this approach in identifying novel targets capable of modifying drinking behaviors.

## 2. Materials and Methods

### 2.1. Subjects

Four Core Genotypes mice were bred at the Laboratory of Animal Resources at Miami University. The original breeding pairs were obtained from Dr. Arthur Arnold at UCLA in 2019 [8–10]. Breeding an XY/Sry+ male with a female C57BL/6J female (Jackson Labs, Bar Harbor, ME) produced four genotypes: XX/Sry−, XX/Sry+, XY/Sry−, and XY/Sry+. The genotypes were acquired by sampling ear tissue and testing was performed by Transnetyx (Cordova, TN).

Prior to the start of drinking, the mice were group-housed by gonad type (*Sry*− or *Sry*+) in a temperature-controlled room. The room was on a 12:12 hour light cycle with lights on at 7:00 AM EST and lights off at 7:00 PM EST. Five days before the beginning of alcohol drinking, mice were housed alone in standard shoebox Udel^®^ Polysulfone rectangular mouse cages of dimensions 18.4 × 29.2 × 12.7 cm. Cages also contained nestlets (5.08 × 5.08 cm) and Bed-O-Cobb bedding (Cincinnati Lab Supply, Cincinnati, OH). Throughout the experiment, the breeder and experimental mice were given *ad libitum* access to Rodent Diet 5001 chow (Cincinnati Lab Supply) and reverse-osmosis (RO) water. Standard guidelines of care set by the National Institutes of Health were followed and all protocols and procedures were pre-approved by Miami University’s Institutional Animal Care Use Committee (IACUC).

### 2.2. Drugs

Ethanol solution (20%) was prepared volume/volume in RO water from a stock solution of 100% ethanol and was made fresh before each drinking session.

### 2.3 Intermittent Ethanol Access

**Figure 1.**
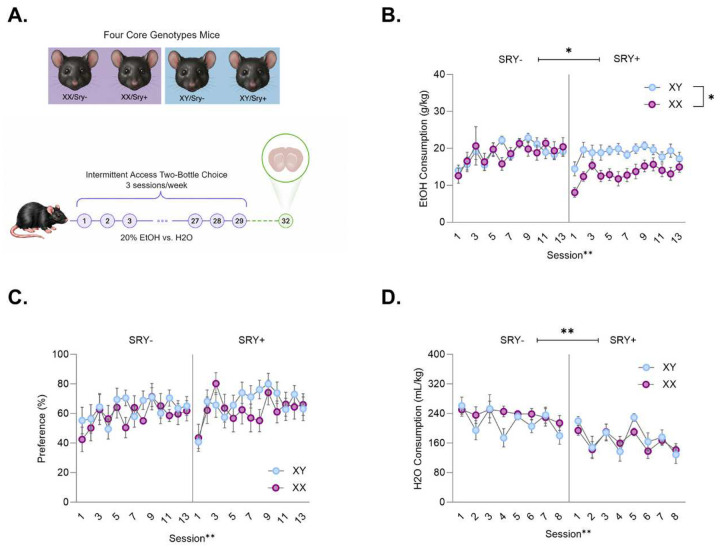
Intermittent Access to Ethanol in Four Core Genotypes (FCG) Mice. **A)** XX and XY mice with testes (*Sry*+) or ovaries (*Sry*−) consumed 20% ethanol vs. water for 13 sessions over 29 days. Tissue was collected from the nucleus accumbens 3 days after the final drinking session. **B)** Ethanol intake was higher in *Sry*− vs. *Sry*+ mice and in XY vs. XX mice with testes. **C)** Preference for ethanol vs. water increased over sessions but was not affected by sex chromosome complement or gonad type. **D)** Water consumption on intervening days was higher in *Sry*− vs. *Sry*+ mice. * p< 0.05, ** p<0.01 (RM two-way ANOVA).

When the experiment began, ethanol-drinking mice (N = 54) were given access to two drinking bottles 6–8 hours after the start of the light cycle. Bottles remained on the cages for 24 hours and were then weighed and replaced 5 days/week. On Mondays, Wednesdays, and Fridays, the bottles contained RO water and 20% ethanol. On Tuesdays and Thursdays, both bottles contained RO water. On Saturdays and Sundays, both bottles contained RO water but consumption was not recorded. This weekly procedure was repeated for a total of 13 ethanol sessions (i.e., 29 days) (Figure 1A). Two cohorts of mice were tested, and the procedure was carried out identically. Every week (3 ethanol sessions), the placement of ethanol and water was switched to reduce side biases. Water-drinking mice were treated the same, with two bottles of RO water presented instead of ethanol. Bottle weights in these mice were recorded once per week on Monday.

Two empty cages containing two dummy bottles each were included as controls for spillage and evaporation. The weights of the mice were taken weekly on Wednesdays immediately prior to the bottle change.

### 2.4 Tissue Collection

Tissue samples were collected three days after the last ethanol drinking session (i.e., day 32). Mice were sacrificed with CO2 followed by cervical dislocation and rapid decapitation. Brains were harvested and flash frozen in 2-methylbutane chilled by dry ice and were then stored in a −80°C freezer until tissue collection. Micro punches (1 mm) of tissue were collected from the NAc core of 1 mm-thick frozen tissue slices. Samples were placed in PCR-clean tubes and stored in a −80°C freezer.

### 2.5 RNA Sequencing

For analysis, samples (N= 21 ethanol- and 21 water-drinking; N = 4–6/genotype) were packaged in dry ice and sent to Novogene (Sacramento, CA, USA). Poly-T oligo attached magnetic beads were used to purify mRNA from total RNA. After fragmentation, the first strand cDNA was synthesized using random hexamer primers, followed by the second strand cDNA synthesis using either dUTP for directional library or dTTP for non-directional library. For the non-directional library, it was ready after end repair, A-tailing, adapter ligation, size selection, amplification, and purification. For the directional library, it was ready after end repair, A-tailing, adapter ligation, size selection, USER enzyme digestion, amplification, and purification. The library was checked with Qubit and real-time PCR for quantification and bioanalyzer for size distribution detection. Quantified libraries were pooled and sequenced on Illumina platforms, according to effective library concentration and data amount.

### 2.6 Gene Expression Analysis

#### 2.6.1 Data quality control.

Raw data (raw reads) of fastq format were first processed through in-house perl scripts. In this step, clean data (clean reads) were obtained by removing reads containing adapter, reads containing poly-N, and low quality reads from raw data. At the same time, Q20 scores, Q30 scores and GC content were calculated from the clean data. All the downstream analyses were based on clean, high-quality data.

#### 2.6.2 Reads mapping to the reference genome.

Reference genome and gene model annotation files were downloaded from the genome website directly. The index of the reference genome was built usingHisat2 v2.0.5 and paired-end clean 1 reads were aligned to the reference genome using Hisat2 v2.0.5. We selected Hisat2 [2] as the mapping tool. Hisat2 can generate a database of splice junctions based on the gene model annotation file and thus provide a better mapping result than other non-splice mapping tools.

#### 2.6.3 Quantification of gene expression level.

featureCounts [3] v1.5.0-p3 was used to count the reads numbers mapped to each gene. Then, FPKM of each gene was calculated based on the length of the gene and reads count mapped to this gene. FPKM (=expected number of Fragments Per Kilobase of transcript sequence per Million base pairs sequenced) considers the effect of sequencing depth and gene length for the reads count at the same time, and is currently the most commonly used method for estimating gene expression levels.

#### 2.6.4 Differential expression analysis.

Differential expression [5] analysis of two conditions/groups (two biological replicates per condition) was performed using the DESeq2Rpackage (1.20.0). DESeq2 provides statistical routines for determining differential expression in digital gene expression data using a model based on the negative binomial distribution. The resulting P-values were adjusted using the Benjamini and Hochberg’s approach for controlling the false discovery rate. Genes with an adjusted P-value (padj)<=0.05 found by DESeq2 were assigned as differentially expressed genes (DEG). DEGs were identified by comparing water to ethanol drinking mice within each of the following groups: male (XY/Sry+), female (XX/Sry−), Sry+, Sry−, XY, and XX. Sex differences in DEGs were further identified by comparing each of the following groups in water and ethanol drinking mice: male (XY/Sry+) vs. female (XX/Sry−), Sry+ vs. Sry−, and XY vs. XX. When analyzing the effects of gonadal status or sex chromosome complement, genotypes were collapsed so all mice with a particular gonadal type or chromosomal complement were included.

#### 2.6.5 Enrichment and functional analysis of differentially expressed genes.

Gene Ontology [7] (GO) enrichment analysis of differentially expressed genes was implemented by the clusterProfiler R package, in which gene length bias was corrected. GO terms with corrected P-value less than 0.05 were considered significantly enriched by differentially expressed genes.

The AI tool Perplexity was used to further assist with identifying the functional significance of DEGs of interest and creating tables. Curated lists of genes were provided alongside prompts such as “An RNASeq analysis was performed using Four Core Genotypes mice. These genes were differentially expressed in the nucleus accumbens of XY mice drinking ethanol vs. water. Analyze for trends based on biological function and behavior and sort into functional themes.”

### 2.7 Drinking data analysis

The consumption for each mouse per session was calculated by subtracting the weight of the dummy bottles from the bottle weight loss (*= (Initial Bottle Weight – Final Bottle Weight) – Average Loss of Dummy Bottles)*. Fluid volumes were then calculated based on the density of a 20% ethanol solution (=0.96864 g/ml) [23] and converted to g/kg by multiplying solution volume by the ethanol fraction and the density of pure ethanol (0.789 g/mL) and normalizing to the weekly body weight. The preference between the two bottles was calculated as = (Volume of Ethanol / (Volume of Ethanol + Volume of Water))*100. Water consumption on the intervening days was calculated from the sum of the corrected weight loss from the two water bottles and expressed as ml/kg of body weight.

There were limited occurrences of spilling bottles or measuring errors; each instance of this was excluded from that session’s calculations. All data reported and displayed shows the mean plus or minus the standard error of the mean. Analysis and figure creation were performed using GraphPad Prism v10 (La Jolla, CA). Drinking data were analyzed using repeated measures (RM) analysis of variance (ANOVA), with the Greenhouse-Geisser correction applied when the assumption of sphericity was violated (ε < 0.75).

## 3. Results

### 3.1 Intermittent Ethanol Drinking.

Drinking over the 13 sessions of intermittent access was assessed using RM three-way ANOVA. For ethanol consumption (Figure 1B), there was a main effect of session (F_6.847, 323.8_ = 6.692, p < 0.001). The main effects of sex chromosome complement (F_1, 50_ = 6.389, p = 0.015) and Sry (F_1, 50_ = 5.582, p = 0.022) were also significant. Finally, there was a significant interaction between sex chromosome complement and Sry (F_1, 50_ = 4.924, p = 0.031). These statistical effects reflect lower ethanol consumption in XX/Sry+ mice than all other groups.

Preference for ethanol vs. water increased over sessions (F_7.647, 380.4_ = 5.156, p < 0.001), but was not affected by Sry or sex chromosome complement (Figure 1C). There were no other significant main effects or interactions involving preference (all p > 0.170).

Water consumption on the intervening session (Tues/Thurs), was greater in *Sry*− mice (F_1, 50_ = 17.140, p < 0.001) and decreased over sessions (F_2.920, 146_ = 8.688, p < 0.001) (Figure 1D). There were no other significant main effects or interactions (all p > 0.08).

### 3.2 Differential Gene Expression in the Nucleus Accumbens.

#### 3.2.1 Water vs. ethanol analyses.

**Figure 2.**
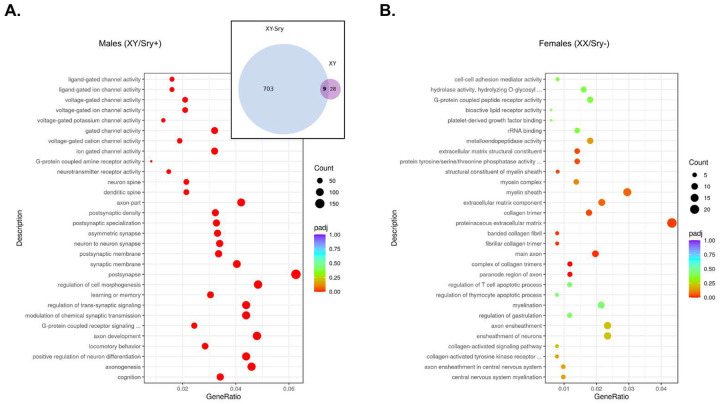
Top 30 terms identified in a Gene Ontology Analysis Between Water- and Ethanol-Drinking Mice. **A)** Male (XY/Sry+) mice displayed robust transcriptional changes in the nucleus accumbens following ethanol drinking (712 differentially expressed genes identified). Thirty-five genes were differentially expressed following ethanol drinking in XY mice, 9 of which overlapped with those identified in XY/Sry+ males (inset). No genes were identified in Sry+ mice. **B)** Only one gene was differentially expressed when comparing water and ethanol drinking female (XX/Sry−) mice.

DEGs were identified by comparing water to ethanol-drinking mice (padj < 0.05). In male (XY/Sry+) mice, 712 DEGs were identified when comparing water vs. ethanol-drinking animals (515 downregulated and 197 upregulated by ethanol). In contrast, only one gene met the statistical threshold to be considered differentially expressed in XX/Sry− females (*Gm10222*). A Gene Ontology (GO) analysis identified the top 30 enriched terms for male (XY/Sry+) (Figure 2A) and female (XX/Sry−) (Figure 2B) mice by statistical significance. Terms enriched following ethanol drinking in males included multiple terms related to synaptic signaling and neural excitability, structural plasticity, and behavioral terms such as “cognition” and “learning and memory.” Although not reflective of statistically significant genes, the top terms for females included those related to the axon, myelination, and the extracellular matrix.

The genes that met the padj threshold for statistical significance in males are listed in **Table 1** and **Table 2**. Statistically upregulated genes in ethanol-drinkers included neurotransmitter receptors, ion channels, and second-messenger pathways required for synaptic signaling (**Table 1**). There was also strong enrichment in genes related to axon guidance, extracellular matrix remodeling, cell adhesion, metabolic responses, and transcriptional programs. The downregulated genes were primarily involved in axon guidance, cell adhesion, neurotransmitter signaling (e.g., glutamate, GABA, acetylcholine, serotonin, neuropeptides, hormones), and metabolic and immune responses (**Table 2**).

When collapsing across sex chromosome complement, there were no differences between water- and ethanol-drinking *Sry*+ mice (i.e., zero DEGs were identified). In *Sry*− mice, five genes were down (*Sstr2*, *Irf2bp2*, *Tns2*, *Ddit4*, *Mcam*) and two were up (*Slc13a5*, *Gm10123*) in ethanol vs. water drinkers, suggesting limited influences of gonadal status alone on gene expression. In XX mice, one gene was up (*Gm10925*) in ethanol- vs. water-drinking mice. In XY mice, two genes were down (*AC151275.1*, *Gm5421*) and 35 genes were up with ethanol. Twenty-eight DEGs in XY mice (2 down and 26 up) were unique and not shared with the DEGs identified for XY/Sry+ male mice (Figure 2A **inset**). The functions of all the 35 upregulated genes identified in XY mice were primarily related to hepatic responses, inflammatory tone, and metabolism/detoxification (**Table 3**).

#### 3.2.2 Male vs. female analyses.

**Figure 3.**
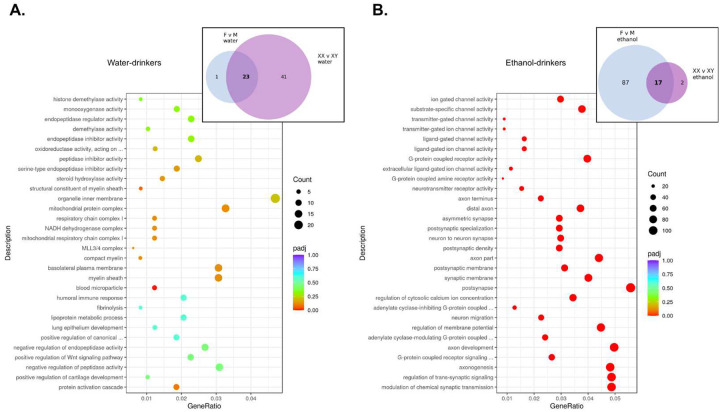
Top 30 terms identified in a Gene Ontology Analysis Between Male and Female Mice. **A)** Comparing male and female mice drinking water identified 24 differentially expressed genes. For XX vs. XY mice, there were 64 genes differentially expressed. **B)** In ethanol-drinkers, 104 genes were differentially expressed between males and females. Nineteen were identified when comparing XX and XY and 0 when comparing *Sry*− to *Sry*+ groups (inset).

Differential gene expression was also analyzed by comparing male and female groups within each drinking solution. There were 24 DEGs between male (XY/Sry+) and female (XX/Sry−) FCG mice drinking water (**Table 4**). This number increased to 104 when mice drank ethanol. A GO analysis identified the top 30 enriched terms by statistical significance for water- (Figure 3A) and ethanol-drinking (Figure 3B) mice. For ethanol-drinking mice, the identified terms were primarily related to channel activity and synaptic signaling, suggesting sex-specific effects of ethanol on these processes in the NAc. After removing genes that were also differentially expressed in water-drinking groups, 70 DEGs were identified that were uniquely upregulated in females vs. males drinking ethanol (**Table 5**). Genes involved in neural excitability and receptor signaling were identified, including excitatory and inhibitory neurotransmitter systems as well as calcium signaling via ion channels and second messenger systems. Other functions of interest were related to circuit remodeling, including cell adhesion and axon guidance. There were 18 unique genes downregulated in females vs. males drinking ethanol, including ones involved in circuit remodeling and immune signaling (**Table 6**).

In both water and ethanol-drinking mice, no DEGs were identified when comparing *Sry*− to *Sry*+ groups. Thus, gonadal status alone did not drive differential expression of any genes. Comparison of XX to XY mice identified differential expression of 64 genes in water-drinking mice and 20 genes in ethanol-drinking mice. For water-drinkers, 23 of these genes were also differentially expressed in XX/Sry− vs. XY/Sry+ mice and 41 were uniquely driven by sex chromosome complement (Figure 3A **inset**). For ethanol drinkers, 17 of the 19 genes were also differentially expressed in XX/Sry− vs. XY/Sry+ mice (Figure 3B **inset**).

## 4. Discussion

This study reveals unique influences of ethanol on gene expression in the NAc of male and female mice that result from the combined influence of gonadal status and sex chromosome complement. Higher ethanol consumption in XY vs. XX male (*Sry*+) mice was observed, replicating previous results using a model of binge-like ethanol consumption [9]. Another key takeaway is that intermittent ethanol consumption drove differential expression of 712 genes in XY/Sry+ males but only one in XX/Sry− female mice, despite similar levels of consumption between the XX/Sry− and XY/Sry+ animals. Thus, female NAc was relatively resistant to the effects of ethanol under these exposure conditions.

We have previously observed effects of sex chromosome complement on alcohol drinking behaviors, with the direction of the effect depending on the behavioral measure. For example, XX mice consumed more alcohol than XY mice under continuous access conditions [8–10], but this pattern was reversed when using a drinking in the dark model of binge drinking [9]. The current results using a 24-h intermittent access home cage drinking task align with the latter task. Thus, when access is limited and/or intermittent, XY chromosome complement (vs. XX) drives increased consumption in males. *Sry*− mice also consumed more than *Sry*+, which supports findings suggesting hormonal effects on drinking [2,5,6,24]. It is important to note that differential intake is a limitation of the current bioinformatic approach, as XX/Sry+ mice received lower levels of ethanol exposure than the other three groups.

To examine the transcriptional response to ethanol, we compared differential gene expression in water vs. ethanol-drinking mice of each sex, gonad type, and sex chromosome complement. The transcriptional response in male (XY/Sry+) mice was robust and suggests that voluntary ethanol drinking recruits processes and pathways involved in neural excitability and signaling, structural and synaptic plasticity, and metabolic adaptation. The identification of these molecular pathways is not particularly surprising considering the large body of literature outlining the involvement of NAc neurotransmitters and neuroplasticity in motivation for ethanol [25–32]. The identification of excitatory and inhibitory neurotransmitter systems, ion channels, and second messenger systems also aligns with the literature on NAc transcriptional responses to ethanol drinking in male rodents [16,17,19,20,33].

What was more striking was the lack of a similar transcriptional response in female (XX/Sry−) mice. Reduced impacts of ethanol on gene expression in females have been observed previously [34,35]. For example, there were more differentially expressed genes in male vs. female hippocampus following postnatal day 7 ethanol exposure in 6 of 8 strains studied [34,35]. Another study in C57BL/6J mice demonstrated greater differential gene expression in males vs. females across several brain regions, including the amygdala, prefrontal cortex, and hypothalamus [34,35]. Most directly relevant to the current experiments, Finn and colleagues completed a focused qPCR analysis of 384 genes in the mouse NAc following binge-like consumption [20]. This approach identified 70 genes regulated by alcohol in female mice, only 14 of which overlapped with the genes identified in males [20]. While the number of DEGs identified in that study did not differ between males and females, the types of genes affected by alcohol consumption did (e.g., neuroimmune functions were affected in females and neurotransmitter metabolism in males). This result agrees with the current findings in that it suggests the response of the NAc transcriptome to ethanol drinking is sexually divergent.

As females are generally considered to be more vulnerable to the physiological impacts of ethanol and addictive behavior [2,24,36], the robust transcriptional response in males may reflect adaptations to ethanol that protect against its harmful effects. For example, downregulation of pathways involved in neural plasticity could confer resistance. Another possibility is that the transcriptional response was driven by stronger withdrawal in males vs. females [37–39]. Indeed, a very different pattern of results could emerge if these analyses were repeated with tissue taken immediately after the drinking session (as in Finn et al., 2018) instead of three days after. However, these results are unlikely to be explained by differences in ethanol metabolism, as male and female mice in this study consumed similar amounts of ethanol relative to their body weights and we have not observed differences in blood ethanol concentration or loss of righting reflex among the four genotypes tested [9]. Future studies of sex differences in gene transcription following ethanol drinking should vary the timing of tissue collection and investigate additional brain regions known to contribute to drinking behaviors.

It is important to keep in mind that changes in transcription levels could have occurred even if a gene failed to meet the statistical threshold to be counted as differentially expressed. Differential expression may have been more variable in females, and thus harder to detect in the water vs. ethanol analysis. By comparing females directly to males in water- and ethanol-drinking mice, we were able to identify a smaller list of genes (N=88) with differential expression driven by sex. As expected from the analysis in males, most of these genes were lower in males vs. females and reflected changes in structural plasticity and synaptic signaling pathways. Of these, *Gpc3* (encodes glypican 3, which is involved in growth factor signaling), *Gpr50* (orphan g-protein-coupled receptor with homology to melatonin receptors), *Aff2* (transcription factor), and *Gprasp2* (G-protein-coupled receptor associated sorting protein 2) may be of interest for sex differences because they are located on the X chromosome.

Despite robust impacts of gonad type and sex chromosome complement on consumption, neither of these factors explained the patterns of gene expression observed in male (XY/Sry+) and female (XX/Sry−) FCG mice. Indeed, gonad type (*Sry*− vs. *Sry*+ comparison) did not produce differential expression of any genes in water or ethanol drinkers and only 7 DEGs were identified when comparing the water and ethanol groups within each gonad type (0 DEGs identified in *Sry*+ and 7 in *Sry*− mice). Of these 7 genes, *Sstr2* (somatostatin receptor 2) is of particular interest for its role in binge drinking and stress [40]. Notably, activation of somatostatin-expressing GABAergic neurons in the nearby bed nucleus of the stria terminalis reduced alcohol consumption in female, but not male, mice [41]. The influence of sex chromosome complement was also limited, although 35 genes were upregulated in XY mice when comparing ethanol to water drinking. The involvement of these genes in liver responses, inflammation, and detoxification may suggest sex differences in the response to ethanol’s neurotoxic effects that are driven by sex chromosome complement. Comparing XX to XY mice revealed three genes that were unique to ethanol drinkers. One of particular interest is *Tlr7*, which is an X-linked gene involved in inflammatory signaling that has been linked to alcohol consumption [42].

## 5. Perspectives and Significance.

Together, these results suggest stark sex differences in the transcriptional response to voluntary ethanol drinking in the mouse NAc. Further, these sex differences result primarily from interactions between gonad type and sex chromosome complement, with a smaller subset of the response attributable to genetic factors. Thus, when considered alongside multiple studies demonstrating effects of sex chromosomes on behavior [8,9,43–45], it is clear that gonadal hormones should not be the only factor considered when studying the mechanisms underlying sex differences.

## Data Availability

The datasets generated and analysed for the current study are available in the Miami University Scholarly Commons repository: https://sc.lib.miamioh.edu/.
